# Multicenter prospective observational study on safety and efficacy of regional citrate anticoagulation in CVVHD in the presence of liver failure: the Liver Citrate Anticoagulation Threshold Study (L-CAT)

**DOI:** 10.1186/cc9547

**Published:** 2011-03-11

**Authors:** T Slowinski, S Morgera, M Joannidis, T Henneberg, R Stocker, E Helset, K Andersen, M Wehner, J Kozik-Jaromin, S Brett, J Hasslacher, JF Stover, H Peters, HH Neumayer, D Kindgen-Milles

**Affiliations:** 1 Charité CCM, Berlin, Germany; 2 Department of Nephrology, Charité CCM, Berlin, Germany; 3 Department of Internal Medicine I, Medical University, Innsbruck, Austria; 4 Department of Visceral and Transplant Surgery, Charité CVK, Berlin, Germany; 5 Surgical Intensive Care, University Hospital, Zurich, Switzerland; 6 Department of Acute Medicine, University Hospital, Oslo, Norway; 7 Clinical Research, Fresenius Medical Care, Bad Homburg, Germany; 8 Department of Anaesthesiology, University Hospital, Duesseldorf, Germany

## Introduction

Regional citrate anticoagulation in continuous venovenous hemodialysis (citrate-CVVHD) has become a widely used technique in the ICU, which decreases risk of bleeding. However, concern exists about safety of citrate in liver failure patients. The aim of our study was to evaluate safety and efficacy of regional citrate anticoagulation in ICU patients with normal and impaired liver function.

## Methods

One hundred and thirty-three consecutive adult ICU patients were prospectively observed for 72 hours of citrate-CVVHD. Patients were stratified into three groups according to their serum bilirubin (mg/dl) (normal: ≤2, *n *= 47, mild: >2 to ≤7, *n *= 44, severe: >7, *n *= 42). Citrate-CVVHD was performed with variable treatment dose using the multiFiltrate device (Fresenius Medical Care, Germany). End-points for safety were: severe acidosis or alkalosis (pH ≤7.2; ≥7.55) and severe hypocalcemia or hypercalcemia (≤0.9; ≥1.5 mmol/l) of any cause. Endpoint for efficacy was the filter lifetime.

## Results

Main types of ICU admission were: 56% medical and 38% post-surgery. Liver failure was predominantly due to ischemia (39%) or multiple organ dysfunction syndrome (27%). The frequency of safety end-points of any cause did not differ between the three patient strata: severe alkalosis (normal: 2%, mild: 0%, severe: 5%; *P *= 0.41); severe acidosis (normal: 13%, mild: 16%, severe: 14%; *P *= 0.95); severe hypocalcemia (normal: 8%, mild: 16%, severe: 12%; *P *= 0.57); severe hypercalcemia (0% in all strata). Only in three patients was an increased ratio of total to ionized calcium (≥2.5) detected (2%). Overall filter lifetime was 49% after 72 hours; however, after censoring for discontinuation due to non-clotting causes (for example, renal recovery, death) 96% of all filters were running after 72 hours.

## Conclusions

Our data demonstrate that citrate-CVVHD can be safely used in patients with liver dysfunction. Furthermore, it yields excellent filter patency and avoids bleeding, and thus can be recommended also in patients with liver dysfunction.

